# Effects of nicotine on microRNA-124 expression in bile duct ligation-induced liver fibrosis in rats

**DOI:** 10.1186/s40360-024-00749-3

**Published:** 2024-03-28

**Authors:** Khalil Hajiasgharzadeh, Parviz Shahabi, Elham Karimi-Sales, Mohammad Reza Alipour

**Affiliations:** 1 Stem Cell Research Center, Tabriz University of Medical Sciences, Tabriz, Iran; 2 Department of Physiology, Faculty of Medicine, Tabriz University of Medical Sciences, Tabriz, Iran

**Keywords:** Liver fibrosis, Bile duct ligation, Nicotine, MicroRNA-124, Inflammation

## Abstract

**Background:**

Nicotine, the main compound of smoking may exert its effects by changing the expression of microRNAs (miRNAs). This study was conducted to further investigate the molecular mechanisms of miRNA-dependent effects of nicotine in an animal model of liver fibrosis.

**Methods:**

The bile duct ligation (BDL) approach was used to create a model of liver fibrosis. Twenty-four male Wistar rats were used in the study. The effects of nicotine administration on miRNA-124 expression, as well as alpha-smooth muscle actin (liver fibrosis marker) and chemokine ligand 2 (an inflammatory chemokine), were investigated using RT-qPCR. In addition, the mRNA and protein expression of signal transducer and activator of transcription 3 (STAT-3; as a potential target for miRNA-124) were investigated by RT-qPCR and immunofluorescence, respectively. Liver enzyme activity levels were measured using a colorimetric assay. In addition, the effects of nicotine on the process of liver fibrosis were investigated with histological studies.

**Results:**

The development of liver fibrosis in BDL rats and nicotine administration led to a decrease in miRNA-124 expression. The decrease in the expression is accompanied by the increase in the expression of fibrotic and proinflammatory genes. Also, an increase in STAT-3 mRNA and protein expression was observed in the fibrotic rats that received nicotine. In addition, the significant increase in bilirubin and liver enzymes in fibrotic rats worsens with nicotine administration. The results of histological studies also confirm these results.

**Conclusion:**

Considering that miRNA-124 is an anti-inflammatory miRNA, it can be concluded that the decrease in its expression due to nicotine exposure leads to an increase in inflammatory processes and subsequently to an increase in liver fibrosis.

## Introduction

The increasing rate of liver fibrosis has become one of the most important health problems worldwide [1]. Liver fibrosis or established cirrhosis is seen in a significant percentage (18–27%) of subjects with risk factors for liver disease [2]. Among the known risk factors of liver fibrosis is smoking and the association between smoking and increased severity of liver fibrosis has been shown in previous studies; however, the exact mechanism of these effects is not known [3, 4]. Previous studies have shown that nicotine, as a fibrogenic compound in cigarette smoke, is involved in several fibrogenic processes in different types of tissues, including the liver [5, 6]. However, the intracellular mechanism of the various effects of nicotine is not clear and there are mounting studies that emphasize the central role of microRNA (miRNA) in creating these effects [7].

miRNAs are ∼ 20–25 nucleotide sequences that play major roles in modifying mRNA expression [8]. Changes in miRNA expression are observed in liver diseases and they are one of the novel therapeutic targets in the diagnosis and treatment of liver fibrosis [9, 10]. Mounting evidence indicates that nicotine leads to alteration in the expression of miRNAs [11]. Among these miRNAs, miRNA-124 is an essential post-transcriptional regulator of inflammatory gene expression [12, 13]. It has been shown in several models of inflammatory diseases that miRNA-124 modulates the expression of inflammatory target genes and participates in the regulation of inflammatory responses [14–16]. Qin et al. conducted a comprehensive review of the role of this miRNA in the immune system and inflammatory diseases, which emphasizes the essential role of this miRNA in regulating immune system activities [17]. In some studies, it has been determined that miRNA-124 can directly target the 3′-untranslated region of signal transducer and activator of transcription 3 (STAT-3) [18]. This transcription factor is one of the major signaling pathways that converts cytokine signals into gene expression programs regulating the immune cells [19]. It is critical to liver diseases, especially in the process of liver chronic inflammation and resulting fibrosis [20]. Considering that chronic inflammation plays an essential role in the pathogenesis of liver fibrosis, it is rational to investigate the role of this anti-inflammatory miRNA-124 [21].

On the other hand, there is some evidence that shows that miRNA-124 is one of the factors influencing the cholinergic anti-inflammatory pathway [22]. The role of inflammatory cytokines and their role in activating hepatic stellate cells and consequently excessive production of extracellular matrix is the main characteristic of liver fibrosis. Therefore, nicotine through the miRNA-124/ STAT-3 pathway may have a possible anti-inflammatory role in liver fibrosis by activating the cholinergic anti-inflammatory pathway. Considering this controversial evidence, this study was conducted to investigate the effects of nicotine on liver fibrosis, focusing on the role of miRNA-124.

## Materials and methods

### Ethics statement

Experiments were performed in compliance with the ethical principles of Tabriz University of Medical Sciences and approved by the Regional Medical Research Ethics Committee (Ethical code: IR.TBZMED.AEC.1402.054). All animal experiments were performed in the animal laboratory of the Department of Physiology, Tabriz University of Medical Sciences.

### Chemicals, animals, and bile duct ligation

In this experiment, 24 male Wistar rats weighing 220–250 g (eight weeks old) were purchased from the animal house of the Tabriz University of Medical Sciences. After adaptation, bile duct ligation (BDL) operation was performed under anesthesia with ketamine and xylazine [23]. In this surgery, the peritoneal cavity was opened, and the bile duct was isolated and triply ligated [24]. The sham-operated rats underwent a similar procedure without ligation of the common bile duct. Then, the animals were randomly divided into four groups of six each: (1) Sham + Saline; (2) Sham + Nicotine; (3) BDL + Saline; (4) BDL + Nicotine. Nicotine (Sigma-Aldrich, product number N3876) was administered intraperitoneally at a dose of 10 mg/kg [25]. Nicotine administration started on the day after surgery and continued every other day for 3 weeks. Previous evidence has demonstrated that nicotine may aggravate the process of liver fibrosis [26–28]. Because of this evidence, we expected to observe the aggravation of hepatic fibrosis in the nicotine-treated groups. Because in previous studies, the severity of liver fibrosis in the BDL model reaches its maximum level after 4 weeks, therefore, we chose a model with the 3-week BDL which exhibits submaximal liver fibrosis and can investigate either protection or exaggeration of hepatic fibrosis in nicotine groups [29]. In this study, death during deep anesthesia with ketamine (100 mg/kg) and xylazine (10 mg/kg) due to opening of the abdomen and chest cavity was considered as a euthanasia method. At the end of the experiments, blood was withdrawn from the heart, and liver tissue was sampled for histology and gene expression analysis. For this purpose, a portion of the liver tissue (middle lobe) was placed in formalin to evaluate histological and immunofluorescence studies, and another portion of the liver tissue (lower part of the right lateral lobe) was placed in -80 refrigerator to measure the expression of target genes.

### RNA extraction and RT-qPCR

The relative miRNA-124 and mRNA levels of alpha-smooth muscle actin (α-SMA), chemokine ligand 2 (CCL-2), and signal transducer and activator of transcription 3 (STAT-3) were assessed by reverse transcription quantitative PCR (RT-qPCR) [30]. All RT-qPCR tests were performed in duplicate from six samples taken from each group (*N* = 6). Total RNA was obtained after Trizol treatment (Invitrogen, Carlsbad, CA); this was performed according to the manufacturer’s guidelines. Total RNA from the liver tissues was obtained and the Prime Script Kit (TaKaRa Bio Inc., Japan) was performed for cDNA synthesis. The purity of extracted RNA was assessed using a NanoDrop Spectrophotometer (Thermo Scientific, USA). The complementary DNA (cDNA) was synthesized using the miScript II RT Kit for miRNA-124 (Qiagen, Hilden, Germany) and α-SMA, CCL-2, and STAT-3 (Biofact, Korea) based on the manufacturer’s guidelines. Then, using SYBR Green fluorescent-based assay, the expression level of miRNA-124 and the mRNA level of target inflammatory genes were determined according to RT-qPCR assay using miScript SYBR Green PCR Kit (Qiagen, Hilden, Germany). Beta-actin was used as a housekeeping gene for target mRNAs, and miR-191-5p was used for normalization of miRNA-124 expression. After normalization, the ratio of expression of each gene was quantified using the 2−(ΔΔCt) method [31]. The primer sequences of the target genes were synthesized and reported in Table 1. All experiments were performed in triplicate.

**Table 1:** The primers sequences for genes

Genes	Sequences		Accession number
rno-miR-124-5p	Target sequence*	5´ CGUGUUCACAGCGGACCUUGAU 3´	MIMAT0004728
rno-miR-191a-5p	Target sequence*	5´ CAACGGAAUCCCAAAAGCAGCUG 3´	MIMAT0000866
CCL-2	Forward	5´ GGGCCTGTTGTTCACAGTTGC 3´	NM_031530.1
	Reverse	5´ GGGACACCTGCTGCTGGTGAT 3´	
STAT-3	Forward	5ˊ-TGGAAGAGGCGGCAGCAGATAGC-3 ´	X91810.1
	Reverse	5ˊ-GCACGGCCCCCATTCCCACAT- 3 ´	
α-SMA	Forward	5´ AACACGGCATCATCACCAAC 3´	NM_031004.2
	Reverse	5´ CACAGCCTGAATAGCCACATAC 3´	
β-actin	Forward	5´ TCACCCACACTGTGCCCCATCTACGA 3´	NM_031144.3
	Reverse	5´ CAGCGGAACCGCTCATTGCCAATGG 3´	

*Sequences were derived from miRBase (www.mirbase.org)

### Serum alanine aminotransferase (ALT, SGPT), aspartate aminotransferase (AST, SGOT), alkaline phosphatase (ALP), and total bilirubin assay

The amount of total bilirubin in the serum and the activity level of liver enzymes, including AST, ALT, and ALP were measured using the colorimetric assay. For this purpose, blood was withdrawn from the heart of nicotine and saline-received sham and BDL rats for serum liver enzyme assay using an auto-analyzer.

### Histological analysis

The liver tissue samples were fixed in formalin and sectioned into 5 μm-thick samples. Histological analysis was performed using hematoxylin and eosin (H&E) as well as Masson trichrome staining. Masson’s trichrome staining is performed for the evaluation of collagen fiber deposition. In this staining approach, the collagen fibers are stained blue, the nuclei will be black and the background will be stained red. In our study, the hepatic inflammation was staged 0–3, with 0 meaning “absent,” 1 meaning “mild,” 2 meaning “moderate,” and 3 meaning “severe [24].”

### Immunofluorescence analysis of STAT-3

Immunofluorescence analysis for STAT-3 was performed as previously described [32]. The primary antibodies were monoclonal anti-STAT-3 (Stat3 (F-2): sc-8019) (diluted 1:100 in PBS, Santa Cruz Biotechnology, Santa Cruz, CA, USA). Fluorescent dye-labeled secondary antibodies were goat anti-mouse IgG H&L (FITC) (diluted 1:200 in PBS; Abcam, Cambridge, MA, USA) and used for detection of primary antibody. The sections were then counterstained with DAPI and were monitored by fluorescence microscopy (Olympus BX50) and evaluated using a DP72 digital camera.

### Statistical analysis

The data were analyzed using GraphPad Prism 6 software (GraphPad Software, La Jolla California, USA) and expressed as Mean ± SEM and analyzed by One-way ANOVA and Tukey’s post-test if normal. Differences between non-normally distributed variables were examined by the Kruskal–Wallis test, and post hoc analysis was performed by using Dunn’s test. The normality of the data was checked using the Kolmogorov-Smirnov test. *P* < 0.05 is considered a statistically significant difference.

## Results

### Confirmation of liver fibrosis development in BDL rats

**Fig. 1:**
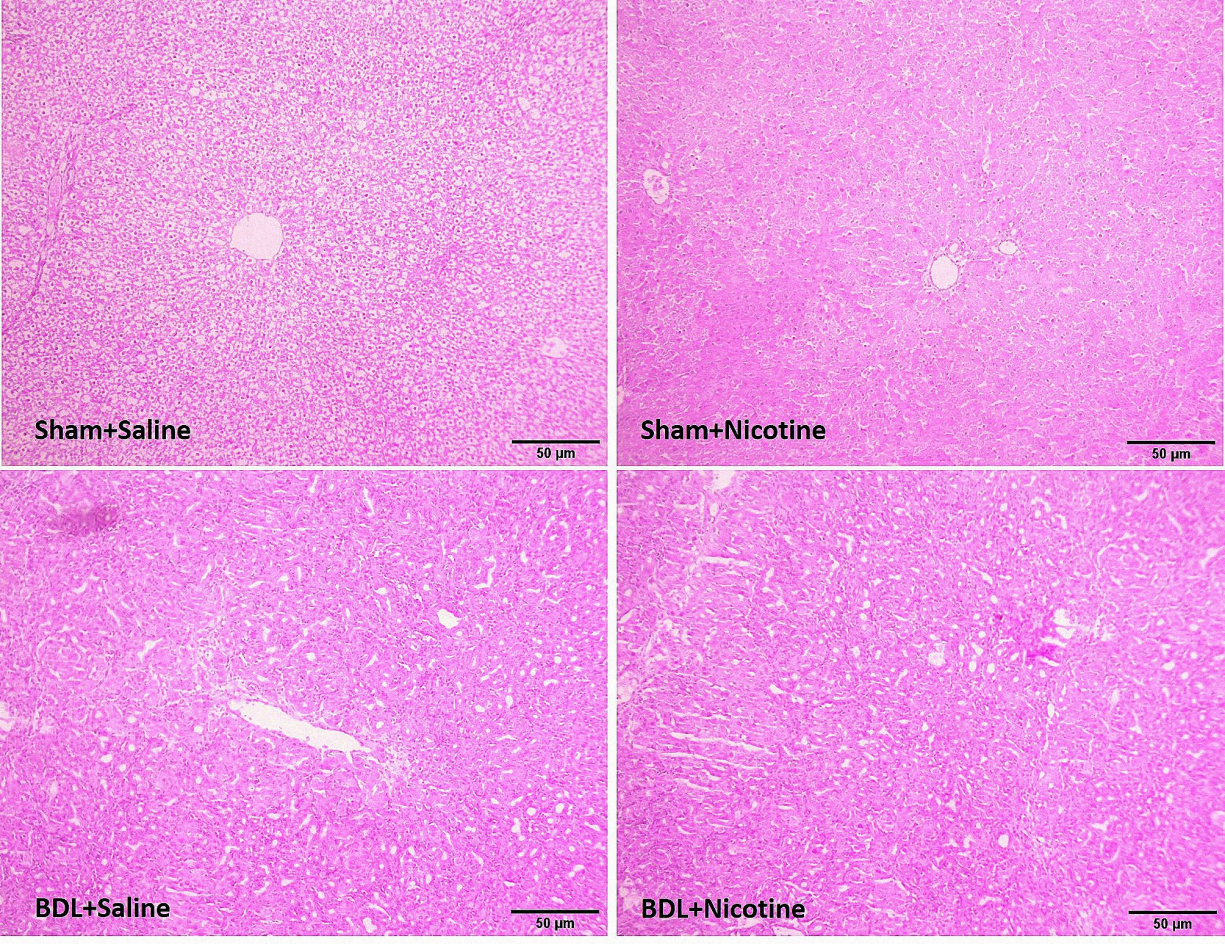
The hematoxylin and eosin stained liver tissues of sham-operated and bile duct ligated rats. The histological structure of sham groups was normal and ductular proliferation and inflammation were absent. The livers of nicotine-treated rats had some degree of portal tract expansion. Portal expansion with the portal-to-portal linkage was the distinctive feature of the bile duct ligated group. Livers of nicotine-treated bile duct ligated rats displayed enhanced degrees of pathological damage and were associated with increased proliferation of biliary epithelia which shows itself as tube-like structures at the periportal region of the liver of nicotine-treated bile duct ligated rats and its intensity was much more in saline-treated bile duct ligated liver samples. All hematoxylin and eosin staining were repeated three times

**Fig. 2:**
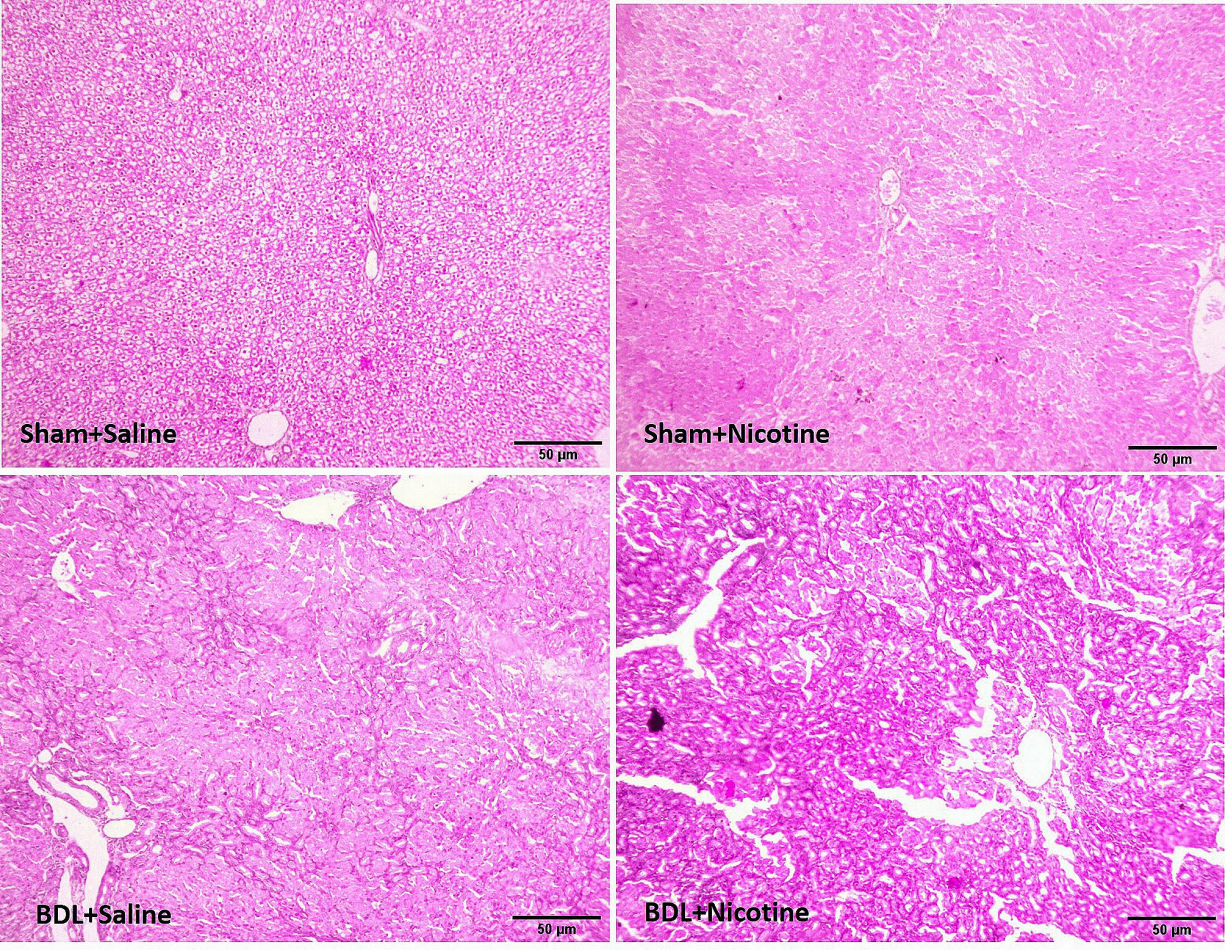
The deposition of collagen fibers was assessed in sham and bile duct ligated rats with and without nicotine. 3 weeks after the surgical procedure, liver tissues were obtained and collagen fibers were stained with Masson’s trichrome. bile duct ligated was associated with extensive bridging fibrosis (portal to portal and portal to central linkage with fibrotic bands). The deposition of fibrotic tissue has increased in the rats of the bile duct ligated group that received nicotine injections. All tests were repeated three times

**Fig. 3:**
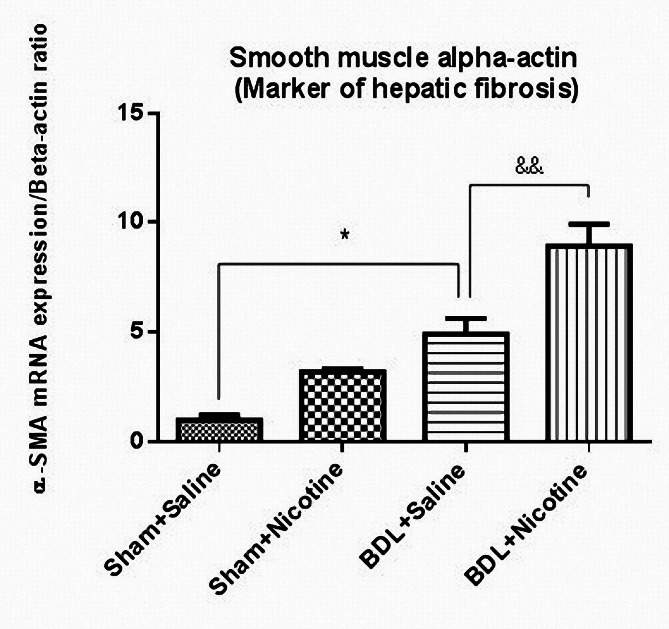
The expression of alpha-smooth muscle actin (α-SMA) in sham and bile duct ligated rats that received normal saline or nicotine about beta-actin as an internal standard. Bile duct ligation has increased this marker of liver fibrosis. *P < 0.05 in comparison with the sham-operated group. Nicotine administration caused a further increase in its expression. && *P* < 0.01 in comparison with the bile duct ligated-saline group. Data are shown as Mean ± SEM and analyzed by one-way ANOVA and Tukey’s post-test. All RT-qPCR tests were performed in duplicate from six samples taken from each group (*N* = 6). The Y-axis represents the relative level of transcriptional difference (alpha-smooth muscle actin/beta-actin)

**Fig. 4:**
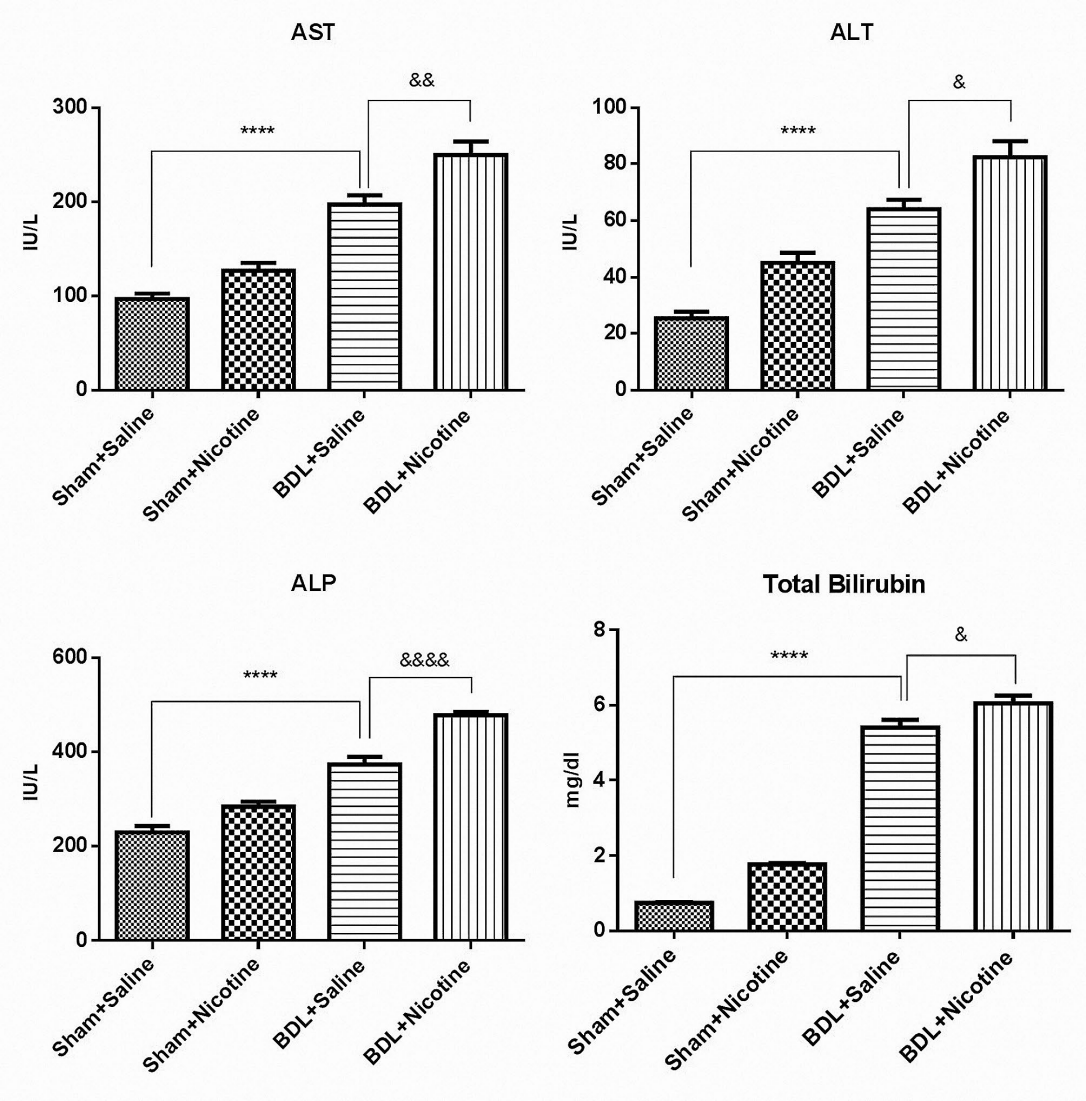
For analysis of liver enzymes and bilirubin levels, 24 male Wistar rats were allocated into the following groups, the Sham + Saline (*N* = 6), Sham + Nicotine (*N* = 6), BDL + Saline (*N* = 6), and BDL + Nicotine (*N* = 6). The effect of 3-week nicotine administration on serum alanine aminotransferase (ALT), aspartate aminotransferase (AST), and alkaline phosphatase (ALP) activities and total bilirubin levels were analyzed in all groups. Data are shown as Mean ± SEM and analyzed by one-way ANOVA and Tukey’s post-test. **** *P* < 0.0001 in comparison with the corresponding sham-operated group. & *P* < 0.05, && *P* < 0.01, &&&& *P* < 0.0001 in comparison with the bile duct ligated-saline group

The induction of liver fibrosis in BDL rats was confirmed by histological H&E (Fig. 1) and Masson’s trichrome staining sections (Fig. 2). In addition, the mRNA level of αSMA gene expression as a marker of hepatic stellate cell activation and liver fibrosis was assessed, and the results confirmed the development of liver fibrosis in BDL rats (Fig. 3). *P* < 0.05 in comparison with the corresponding Sham-Saline group. Also, the results of measuring the total bilirubin concentration in the serum and the activity of liver enzymes showed that these enzymes, which are a marker of liver damage, were significantly increased in the group of fibrotic BDL rats (Fig. 4). All these measures related to histology experiments, the upregulated expression of liver fibrosis marker (αSMA), and elevation of liver enzyme activities have a good degree of validity and showed the development of liver fibrosis in rats.

### Effect of nicotine administration on miRNA-124 expression in BDL rats

**Fig. 5:**
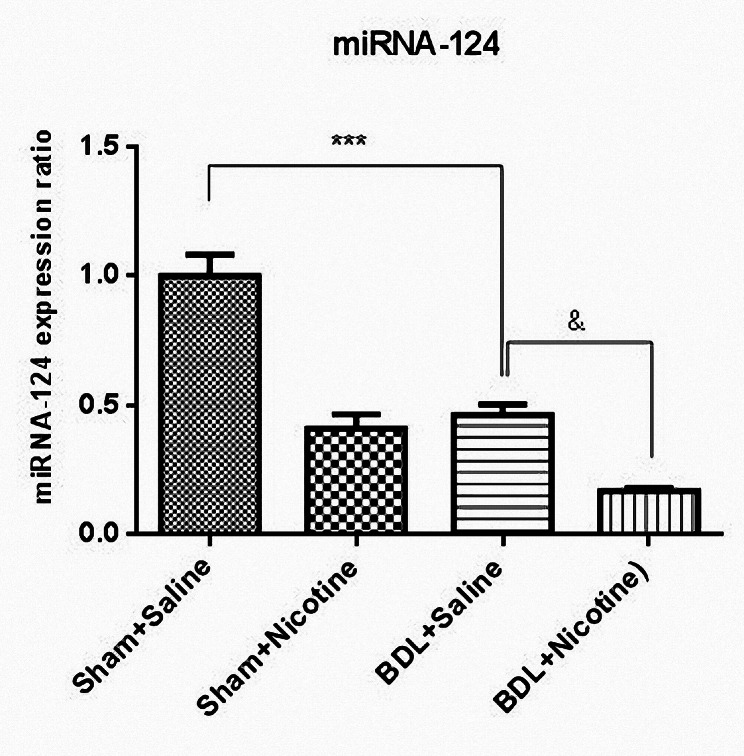
Sham and bile duct ligated rats were administrated with nicotine (10 mg/kg) for 3 weeks, and then the expression level of miRNA-124 was evaluated using RT-qPCR assay. *** *P* < 0.001, in comparison with the corresponding sham-operated group. & *P* < 0.05 in comparison with the bile duct ligated-saline group. Results are expressed as the Mean ± SEM of six experiments (*N* = 6, duplicate determinations) and analyzed by one-way ANOVA and Tukey’s post-test

The obtained results showed that the induction of biliary fibrosis in BDL rats leads to a decrease in miRNA-124 expression (Fig. 5). Nicotine administration for 3 weeks in healthy rats leads to a significant decrease in the expression of this miRNA. The important results of our study were obtained when the BDL rats were administered nicotine. The results showed that the administration of nicotine in the BDL + nicotine group led to a further decrease in the expression of miRNA-124 compared with the BDL + saline group (&*P* < 0.05) (Fig. 5). Therefore, because miRNA-124 is an anti-inflammatory miRNA, in the continuation of the study, we investigated the effects of nicotine administration and reduced expression of miRNA-124 during the development of liver fibrosis.

### Effect of nicotine administration on the infiltration of inflammatory cells and expression of inflammatory genes

**Fig. 6:**
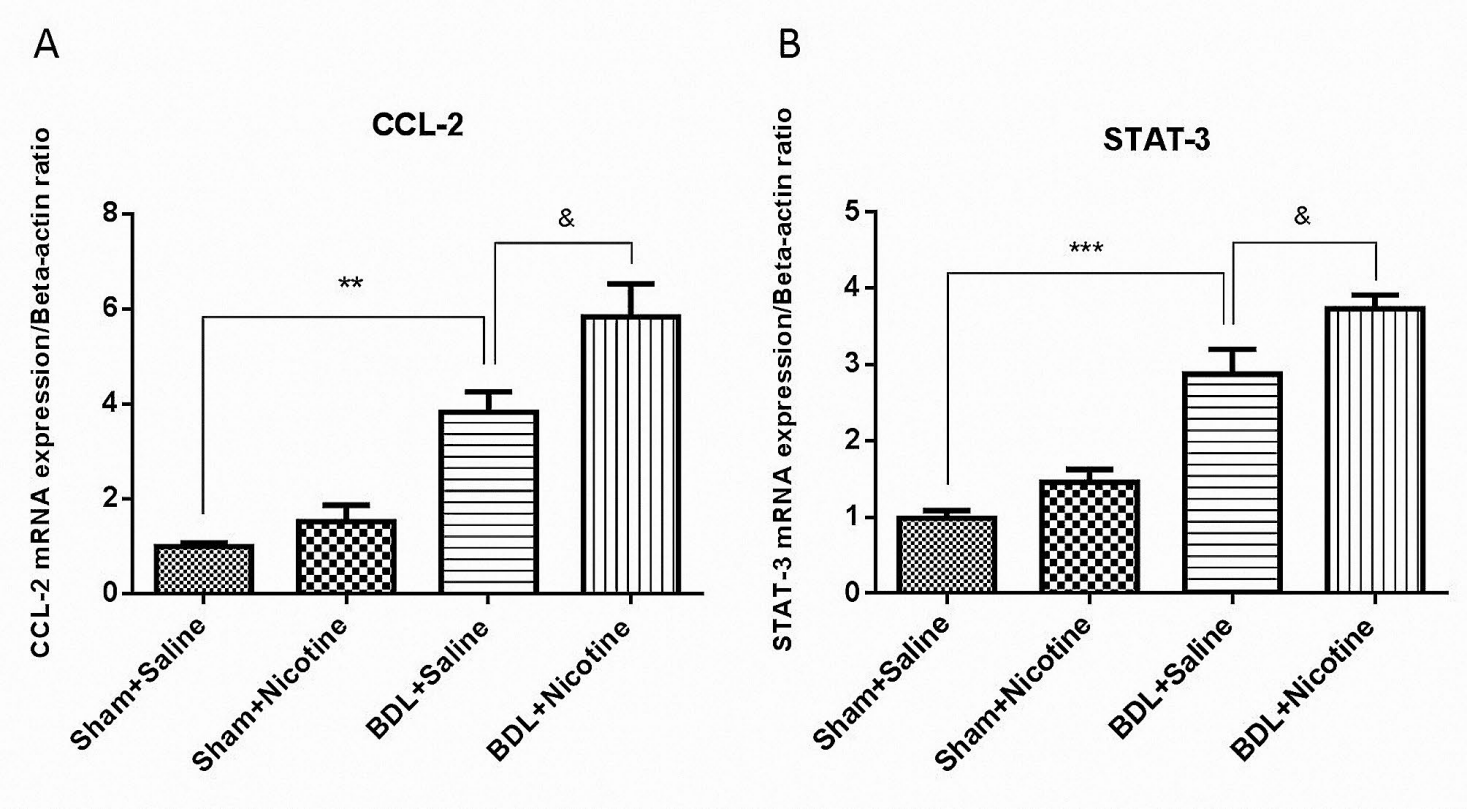
The nicotine or saline was treated in sham or bile duct ligated rats and then the expression level of chemokine ligand 2 (CCL-2) was evaluated using RT-qPCR assay. ** *P* < 0.01, in comparison with corresponding sham-operated group and & *P* < 0.05 in comparison with the bile duct ligated-saline group. Also, the effects of bile duct ligation, as well as administration with nicotine (10 mg/kg) on the mRNA expression of signal transducer and activator of transcription 3 (STAT-3), were evaluated using RT-qPCR assay. Data are shown as Mean ± SEM and analyzed by one-way ANOVA and Tukey’s post-test. *** *P* < 0.001 in comparison with corresponding sham-operated group and & *P* < 0.05 in comparison with the bile duct ligated-saline group (*N* = 6, duplicate determinations)

**Fig. 7:**
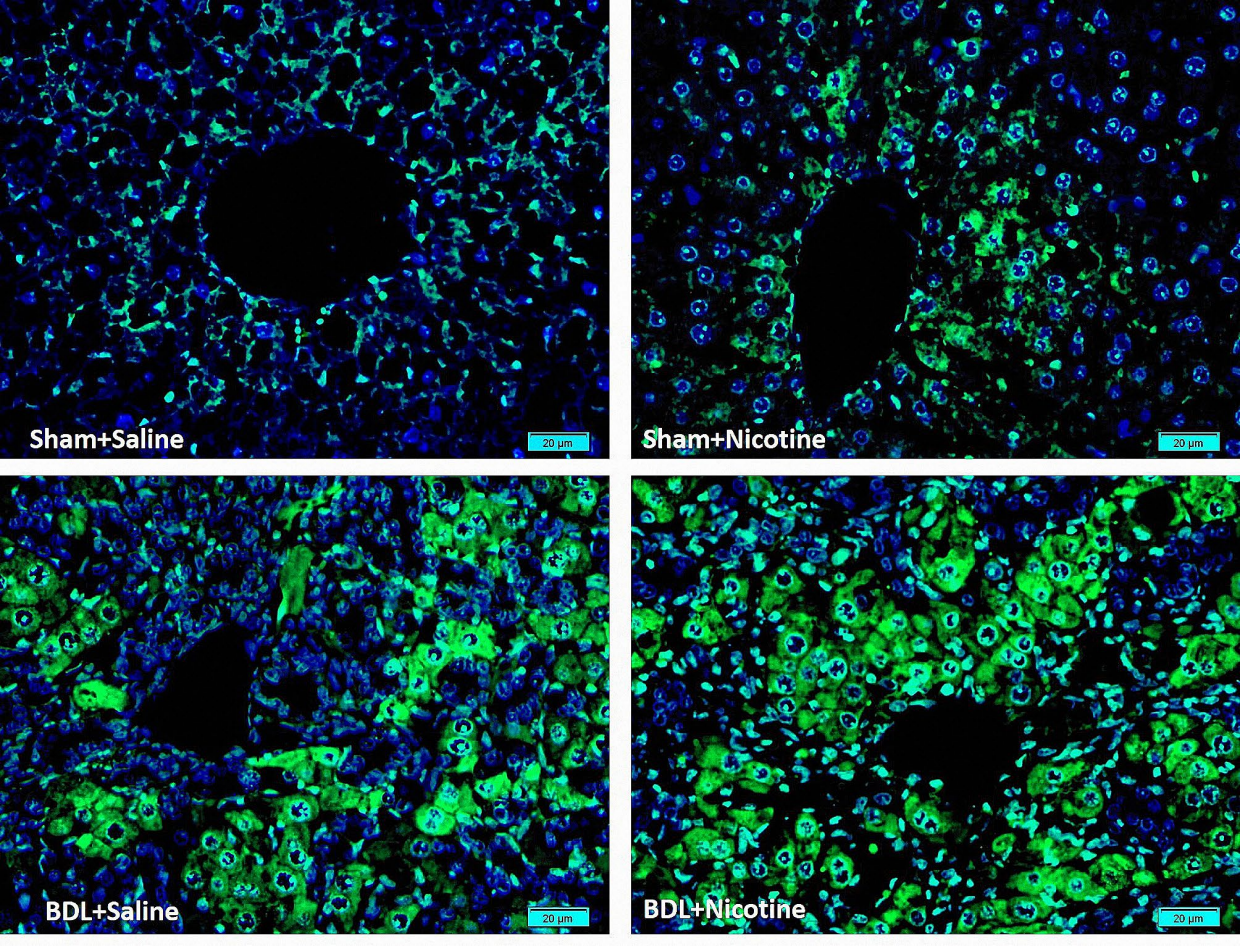
Immunohistofluorescent study using an anti-STAT-3 antibody (green) in a representative rat liver obtained from normal saline or nicotine-treated sham or bile duct ligated rats. Nuclei were stained with DAPI (×400 magnification). Bile duct ligation-induced liver fibrosis leads to increased protein expression of STAT-3 in the liver. The liver of fibrotic rats exposed to nicotine demonstrated an increased expression of STAT-3.

Chronic liver injury leads to the secretion of cytokines such as CCL-2, which are responsible for the infiltration of inflammatory cells in the liver [33, 34]. The effect of nicotine administration on the expression of CCL-2 in rat liver was assessed by using RT-qPCR. The results showed that CCL-2 expression was significantly increased in BDL rats in comparison with sham-operated rats. Nicotine administration in bile duct ligated rats (BDL + Nicotine) further induced the expression level of CCL-2 (& *P* < 0.05) compared with the bile duct ligated-saline group (Fig. 6A). It appears that CCL-2 upregulation is associated with nicotine-induced liver fibrosis in BDL rats. CCL-2, also known as monocyte chemoattractant peptide-1 (MCP-1), acts as a cytokine that leads to an influx of inflammatory cells in the injured tissues [34]. Our histological studies confirmed that there was an infiltration of inflammatory cells in the fibrotic BDL rats, which is increased in the liver of rats receiving nicotine (Table 2). On the other hand, given that previous studies have shown that STAT-3 is one of the specific targets of miRNA-124, we investigated the changes in the expression of STAT-3 in the liver of fibrotic rats receiving nicotine. The results showed that the expression of this gene increases during the development of liver fibrosis, and this increase is even greater in the group of nicotine-received fibrotic rats (Fig. 6B). To determine whether STAT-3 is expressed at the protein level, fluorescent-based staining was performed using an antibody against STAT-3. Figure 7 indicates that in the BDL model, STAT-3 expression increased compared with that in the sham-operated groups. Scattered STAT-3 staining was observed in the hepatocytes of liver sections from sham-operated rats. We observed strong STAT-3 immunostaining in BDL + Nicotine liver samples in comparison with BDL + Saline groups in the immunofluorescent study (Fig. 7). The results show that although bile duct ligation-induced liver fibrosis has led to an increase in STAT-3 expression, nicotine administration leads to an augmented increase in its expression, which is consistent with the results of mRNA expression analysis.

**Table 2:** Histological assessment of hepatic inflammation. Nicotine administration caused a further increase in infiltration of inflammatory cells in BDL + Nicotine group as compared with BDL + Saline group

	Sham + Saline (*n* = 6)	Sham + Nicotine (*n* = 6)	BDL + Saline (*n* = 6)	BDL + Nicotine (*n* = 6)
**Inflammation**				
Score 0	*6*	*2*	*0*	*0*
Score 1	*0*	*4*	*6*	*0*
Score 2	*0*	*0*	*0*	*6*
Score 3	*0*	*0*	*0*	*0*
Median	0	1	1	2 *

* Chi-square test was used to compare between groups

### Effect of nicotine administration on liver enzymes in BDL rats

Serum bilirubin levels and AST, ALT, and ALP activity of BDL rats are shown in Fig. 4. BDL was associated with hyperbilirubinemia and increased ALT, AST, and ALP levels (*P* < 0.0001). These increased liver enzymes worsened when nicotine was administered, indicating more liver damage in the nicotine group (Fig. 4).

### Effect of nicotine exposure on liver fibrosis

The histology of liver fibrosis was studied using H&E and Masson’s trichrome staining (Figs. 1 and 2). BDL was associated with bile duct proliferation (Fig. 1) and extensive bridging fibrosis (portal to portal and portal to central linkage with fibrotic bands) (Fig. 2). As shown in these figures, nicotine has a significant effect on hepatic fibrosis and portal expansion in BDL rats. Likewise, nicotine in sham-operated rats altered the histological structure of the liver as assessed by H&E and trichrome staining. Also, the increased level of the liver fibrosis marker (αSMA) in the fibrotic groups is increased when nicotine is administrated, which indicates higher levels of activation of hepatic stellate cells and more production of extracellular matrix (Fig. 3). These results indicate the adverse effects of nicotine on the severity of liver fibrosis development. To date, no study has investigated the effects of nicotine administration on the development of liver fibrosis in the BDL model, and the results of this study clearly showed that nicotine has a wide range of effects in both liver damage and liver inflammation, as well as the development of liver fibrosis.

## Discussion

Today, changes in lifestyle and the use of fast foods and high-fat foods have increased the prevalence of liver diseases worldwide [35]. According to global statistics, two million people die each year due to chronic liver disorders, and these conditions are responsible for 4% of all deaths [36]. Genetic background, consumption of a high-fat diet, viral infection, and autoimmune diseases are the most common causes of chronic liver diseases. Among these risk factors, some studies have mentioned smoking [37]. Wijarnpreecha et al. found that smoking is associated with a significantly higher risk of advanced liver fibrosis among patients with primary biliary cholangitis [3]. The association between smoking and increased severity of liver fibrosis has been reported in other studies [38–40]. Although various studies have been conducted for many years in the field of understanding the mechanism of liver tissue damage in liver disorders, unfortunately, the main mechanism of liver tissue damage in liver disorders, especially liver fibrosis, has not been fully understood yet. Undoubtedly, knowing the exact mechanism of this damage can be effective in treating and reducing the liver complications of patients with liver fibrosis.

The main goal of this study was to investigate the role of nicotinic acetylcholine receptors (nAChR) in the liver. These receptors are expressed in the liver, but their exact protective or pathological functions remain to be determined. Several studies have been conducted to investigate the expression and function of these receptors in physiological and pathological conditions in the liver. For instance, Sakata et al. investigated the expression of the α7 subtype of nAChR (α7nAChR) in humans and observed the highest accumulation of these receptors in the liver [41]. These observations highlight the potential involvement of α7nAChR in the modulation of liver function. Ehrlich et al. indicated that the expression of α7nAChR was observed in the liver and increased following BDL [42]. Our unpublished data also confirmed that the expression of several subtypes of nicotinic receptors (e.g. α4nAChR, α7nAChR) increases during the BDL-induced liver fibrosis. Also, we have previously investigated the role of inhibiting the activity of these receptors both physiologically (through vagotomy surgery) and pharmacologically (using Methyllycaconitine; an antagonist of α7nAChR) [24]. But in that study, not only did inhibiting the activity of these receptors not affect the progression of liver fibrosis, but also caused a decrease in liver damage and reduced the serum level of liver enzymes. Several studies suggest that α7nAChR is probably a target for the effect of nicotine on the liver [5, 26]. Therefore, in the present study, we used nicotine as an agonist of these receptors to examine the effects of their activation. The obtained results were in line with our previous studies and showed that the activation of nicotinic receptors in the liver leads to the deterioration of the progression of BDL-induced liver fibrosis.

Considering these previous results and the relationship between the miRNA-124-dependent function α7nAChR [22], we continued our studies by investigating the expression of this miRNA under conditions of BDL-induced liver fibrosis and nicotine administration. The obtained results showed that the process of liver fibrosis in BDL rats leads to a decrease in the expression of miRNA-124. Another finding in our previous experiments was the investigation of the interaction between miRNA-124 and the target genes. In that study, using the Luciferase assay, our team showed that the STAT-3 gene was one of the target genes of this miRNA [18]. We can perform in vitro miRNA transfection, and in our previous study, we investigated the effects of transfection with this miRNA-124. The obtained results showed that the decrease in the expression of miRNA-124, which occurs due to exposure to nicotine, is accompanied by the increase in the expression of STAT-3, which is consistent with the previous results obtained by luciferase assay [18]. Also, to confirm the results at the protein level, we performed the immunofluorescence test and observed that the liver of fibrotic rats exposed to nicotine showed an increased expression of STAT-3. We showed that BDL-induced liver fibrosis leads to increased mRNA and protein expression of STAT-3 in the liver. These results are consistent with the results related to the deterioration of liver structure and function in the condition of liver fibrosis and nicotine administration.

Many studies emphasize the pivotal role of miRNAs in the pathogenesis of liver fibrosis, and therapeutic approaches based on restoring the expression of altered miRNAs have been introduced as new methods of treating liver fibrosis [43, 44]. One of the important miRNAs with anti-inflammatory activities shown in previous studies is miRNA-124. Considering these anti-inflammatory features, changes in the expression level of this miRNA during liver fibrosis can have many consequences. On the other hand, nicotine has been shown in many studies to exert its effects through miRNA pathways. The results showed that nicotine seems to stimulate the activity of the STAT-3 signaling pathway through miRNA-124-related effects. Similar to nicotine, which in this study was associated with an increase in the severity of liver fibrosis, another study, showed that the use of heroin was also associated with an increase in liver fibrosis [45]. We have previously shown that selective hepatic vagotomy leads to a decrease in AST activity in the serum of BDL rats [24]. In the present study, instead of inhibiting the activity of nicotinic acetylcholine receptors, we used the stimulation of these receptors by injecting nicotine. The results we obtained agree with our previous study and show that the stimulation of nicotinic acetylcholine receptors leads to increased damage to the liver, which performs these effects to some extent by reducing the expression of miRNA-124. Therefore, exposure to nicotine or increased activity of nicotinic receptors through endogenous or exogenous ligands can lead to increased liver damage and subsequent liver fibrosis. However, this preliminary study requires additional experiments to prove the causal link between nicotine exposure, miRNA-124 expression, STAT-3 activation, and the induction of inflammatory processes are required.

## Conclusion

Nicotine as a ligand of nAChRs imitates the actions of ACh by binding as an agonist to these receptors. This compound, in part through signaling via nAChRs, plays a crucial role in regulating essential cellular functions and may have critical regulatory roles in the pathogenesis of complicated diseases such as fibrosis. Nicotine exposure can result in various adverse effects on organs that have no direct contact with smoke, such as the liver. The study’s results clearly demonstrate that nicotine exposure leads to an increase in liver fibrosis severity. As such, investigating the role of anti-inflammatory miRNA-124 expression in liver fibrosis processes is crucial, and restoring miRNA-124 expression can be considered a therapeutic approach. It can be concluded that avoiding exposure to nicotine should be taken seriously in people who are at a high risk of developing liver fibrosis.

## Data Availability

The data that support the findings of this study are available from the corresponding authors upon reasonable request.
